# An Approach to Leadership Development and Patient Safety and Quality Improvement Education in the Context of Professional Identity Formation in Pre-Clinical Medical Students

**DOI:** 10.1177/23821205231170522

**Published:** 2023-05-08

**Authors:** Hamza Inayat, Jacqueline Torti, Juliya Hemmett, Lorelei Lingard, Brandon Chau, Ali Inayat, Jason L. Elzinga, Nabil Sultan

**Affiliations:** 1 Schulich School of Medicine & Dentistry at Western University in London, Ontario, Canada; 2 Department of Medicine, and Scientist, Centre for Education Research and Innovation, 70384 Schulich School of Medicine and Dentistry, Western University, London, Canada; 3 Division of Nephrology, Department of Medicine, Cummings School of Medicine, Calgary, Canada; 4 Department of Emergency Medicine, University of British Columbia, Kelowna, Canada; 5 Medical Student at the St. George's University, Grenada, West Indies, and Northumbria University, Newcastle, England; 6 Physician for the Department of Emergency Medicine at the University of Calgary, Calgary, Canada; 7 Nephrologist and Associate Professor in the Department of Nephrology, 70384 Schulich School of Medicine & Dentistry, Western University, London, Canada

**Keywords:** leadership development, professional identity, undergraduate medical education, patient safety and quality improvement

## Abstract

**Objectives:**

Leadership and patient safety and quality improvement (PSQI) are recognized as essential parts of a physician's role and identity, which are important for residency training. Providing adequate opportunities for undergraduate medical students to learn skills related to these areas, and their importance, is challenging.

**Methods:**

The Western University Professional Identity Course (WUPIC) was introduced to develop leadership and PSQI skills in second-year medical students while also aiming to instill these topics into their identities. The experiential learning portion was a series of student-led and physician-mentored PSQI projects in clinical settings that synthesized leadership and PSQI principles. Course evaluation was done through pre/post-student surveys and physician mentor semi-structured interviews.

**Results:**

A total of 108 of 188 medical students (57.4%), and 11 mentors (20.7%), participated in the course evaluation. Student surveys and mentor interviews illustrated improved student ability to work in teams, self-lead, and engage in systems-level thinking through the course. Students improved their PSQI knowledge and comfort levels while also appreciating its importance.

**Conclusion:**

The findings from our study suggest that undergraduate medical students can be provided with an enriching leadership and PSQI experience through the implementation of faculty-mentored but student-led groups at the core of the curricular intervention. As students enter their clinical years, their first-hand PSQI experience will serve them well in increasing their capacity and confidence to take on leadership roles.

## Introduction

The strong association between effective leadership and ensuring safety and quality in complex systems has long been recognized in business and industry, where teamwork and communication are essential.^1–3^ Leadership has similarly been recognized as essential to patient safety and quality improvement (PSQI) in complex healthcare systems.^4,5^ Although leadership and PSQI are acknowledged as core elements of a physician's role and identity;^6–8^ medical students are not given sufficient opportunity to learn these in medical school.^9,10^ As such, students leave the program unprepared for the leadership and PSQI responsibilities they require as residents.

Enabling medical students to form part of their professional identity around being a leader and a steward of the healthcare system offers an important contribution to the next generation of physicians.

We aimed to develop leadership and PSQI skills in pre-clinical second-year medical students through the Western University Professional Identity Course (WUPIC) innovation. We sought not only to build knowledge, skills, and attitudes in these important areas but also to instill leadership and PSQI into the identities of the trainees through experiential learning and mentorship. The intervention aimed to address key challenges of developing leadership and PSQI in a pre-clinical undergraduate setting.

## Methods

### Planning

A course planning committee was struck in 2016 at the Schulich School of Medicine, Western University, and included faculty members of various medical and surgical specialties, educational experts, school administrators, and student representatives. Four major themes were identified for course content: leadership, medical ethics, healthcare system design, and PSQI. The course planning committee launched a course in the 2017/2018 school year following principles from both the LEADS healthcare leadership framework (https://leadscanada.net), a leadership framework outlining a comprehensive approach to leadership development for the Canadian health sector, and the Institute for Healthcare Improvement (IHI) (www.ihi.org), a global body that focuses on developing practical methods to improve the safety of care.

### Delivery of course content

This content was delivered through various teaching modalities, including didactic lectures, small group learning, panel discussions, independent learning, and group projects. Three to four hours weekly were allocated for course content over nine months of the school year. Selected topics for the didactic portion of the course included medical ethics, healthcare financial awareness, effective leadership techniques, healthcare systems, project management, conflict resolution, and team dynamics. Small group sessions focused on discussing challenging clinical scenarios related to organizational and team leadership.

Medical ethics training was designed to build upon pre-requisite knowledge with focused topics on organizational ethics, quality care as an ethical responsibility, social accountability, and end-of-life care. Topics were delivered through a combination of large-group and small-group discussions. A panel of local physicians engaged in community projects discussed ethical implications of their work and principles of social accountability. Classroom teaching was complemented with a peer-to-peer teaching seminar and personal essays exploring principles discussed in class.

The healthcare systems portion of the course consisted primarily of didactic lectures focused on health policy, financing, and auxiliary medical organizations. These didactic sessions served as the basis for students to understand the system they would engage with during their clinical projects. Knowledge was assessed with a multiple-choice exam at the end of the module. Healthcare organization was then explored practically during the QI projects and reflected upon in writing activities at the end of the course. Didactic sessions were presented either by the course coordinators or guest speakers who were content experts in the topics being presented. The overall course was graded on a pass/fail basis. The students were required to pass each section of the course to receive an overall pass, and any failed or missing sections required a remediation plan. The submitted writing activities were not graded beyond pass/fail. Class participation, while highly encouraged, was not specifically graded.

During the first 3 months of the course, students were also asked to complete a foundational PSQI course through the open-access IHI Basic Certificate online program. An evaluation was built into the modules, and students were provided with a certificate upon completion. This independent learning was complimentary to didactic sessions which expanded upon key points and allowed student questions to be addressed.

### Experiential learning-leadership development through PSQI projects

The cornerstone of the course was a series of student-led and physician-mentored PSQI projects that synthesized leadership and PSQI principles using experiential learning in the healthcare setting. Students were placed into project groups (four to five students each) and matched with a physician mentor in the clinical area of interest. Groups discussed with their mentors’ gaps within the clinical area of the mentor. From the discussions, groups brainstormed possible interventions to address these gaps. One project idea was selected to proceed for the course. The projects were rooted in solving real clinical problems. Mentors did not require previous PSQI experience but were provided with an orientation session that outlined their roles. Mentors were physicians working in academic centers, and served as advisors to the groups, orienting students to specific clinical areas in medicine, connecting their groups to key stakeholders, and identifying problems or areas for improvement. Students and mentors were further supported with their projects by four faculties with PSQI expertise. The project was designed to integrate the PSQI process and healthcare system design knowledge with practical experience leading and working within a team. Once a gap was identified, groups brainstormed a proposed intervention and led a PSQI design process through methods learned during the course. Throughout the project, students were required to reflect on the ethical implications of the problems they addressed and the barriers experienced when working toward systemic change. Students scheduled monthly meetings with their mentors to update them on progress and discuss challenges. However, the leadership and design of the projects remained the students’ responsibility.

Course assessment consisted of a written report and an oral presentation. Implementing student change ideas was not necessarily required across all groups but was encouraged when possible. Students presented their progress during a mid-course assessment to receive formative feedback and refine their final project. At the end of the course, student groups presented their final PSQI project to the class. Group presentations and final written reports were evaluated by faculty leads with PSQI expertise based on a standardized evaluation rubric. For the purpose of the course, the PSQI project was considered “complete” once the group presentation was given and the group written report submitted and received a pass on the rubric-based evaluation. Table 1 shows a list of projects completed during the course in 2017/2018.

**Table 1.** List of projects undertaken during the WUPIC 2017/2018 cycle.

Improving End of Life Care of COPD Patients by Increasing Advanced Care Planning Documentation.
Improving advanced care planning discussions in dialysis patients at University Hospital LHSC.
Creating a Standard Pain Assessment Tool to Increase Pain Management For Hemodialysis Patients.
Hyperkalemia in the Paediatric Emergency Room.
Improving Patient Satisfaction with their Inflammatory Bowel Disease Care.
Improving appointment attendance of patients who inject drugs at the outpatient infectious disease clinic in St. Joseph's Health Care, London, ON.
Reducing Antipsychotic Drug Polypharmacy in the LHSC Adult Inpatient Mental Health Service.
Reducing Inappropriate Admissions to Psychiatric Services in Patients with a Primary Medical Illness Complicating their Presentation.
Lowering the transfusion rate in patients with hemodynamically stable iron deficiency anemia in the LHSC emergency department.
Improving primary care access for patients with substance use disorders.
Colorectal Cancer Screening in Family Medicine: a Quality Improvement Project.

Following ethics approval, the course was evaluated using student surveys and semi-structured interviews with mentors for the 2016–2017 academic year. Student survey and interview guide can be found in Supplementary files 1 and 2. Before and after the course, all second-year medical students (188) at the Schulich School of Medicine, Western University were invited to fill out a self-efficacy rating tool using a 7-point Likert scale developed to reflect the course's objectives. The self-efficacy rating tool was developed specifically based on the course objectives. The inclusion criteria included any student enrolled in the second-year WUPIC course and willing to participate in the study. There were no exclusion criteria. Numerical data were analyzed descriptively, and comparisons were made using Student's T-tests. All mentors were invited to participate in semi-structured interviews through email to explore their perceptions of the course and its learning outcomes. Individual interviews took place in private offices and were conducted by JH (MD), BC, and JE (Medical Students). The interviews were audio-recorded and sent to a professional agency for transcription. Returned transcripts were reviewed for accuracy and imported to NVivo (QRS International Pty Ltd, Burlington, Massachusetts, United States), a qualitative data analysis software, and analyzed by three members of the research team (J. M. I. T., A. I., and H. I.). The qualitative data were analyzed through an inductive content analysis,^9^ and the COREQ (Consolidated Criteria for Reporting Qualitative Research) Checklist was completed (supplementary 3).

## Results

**Figure 1.**
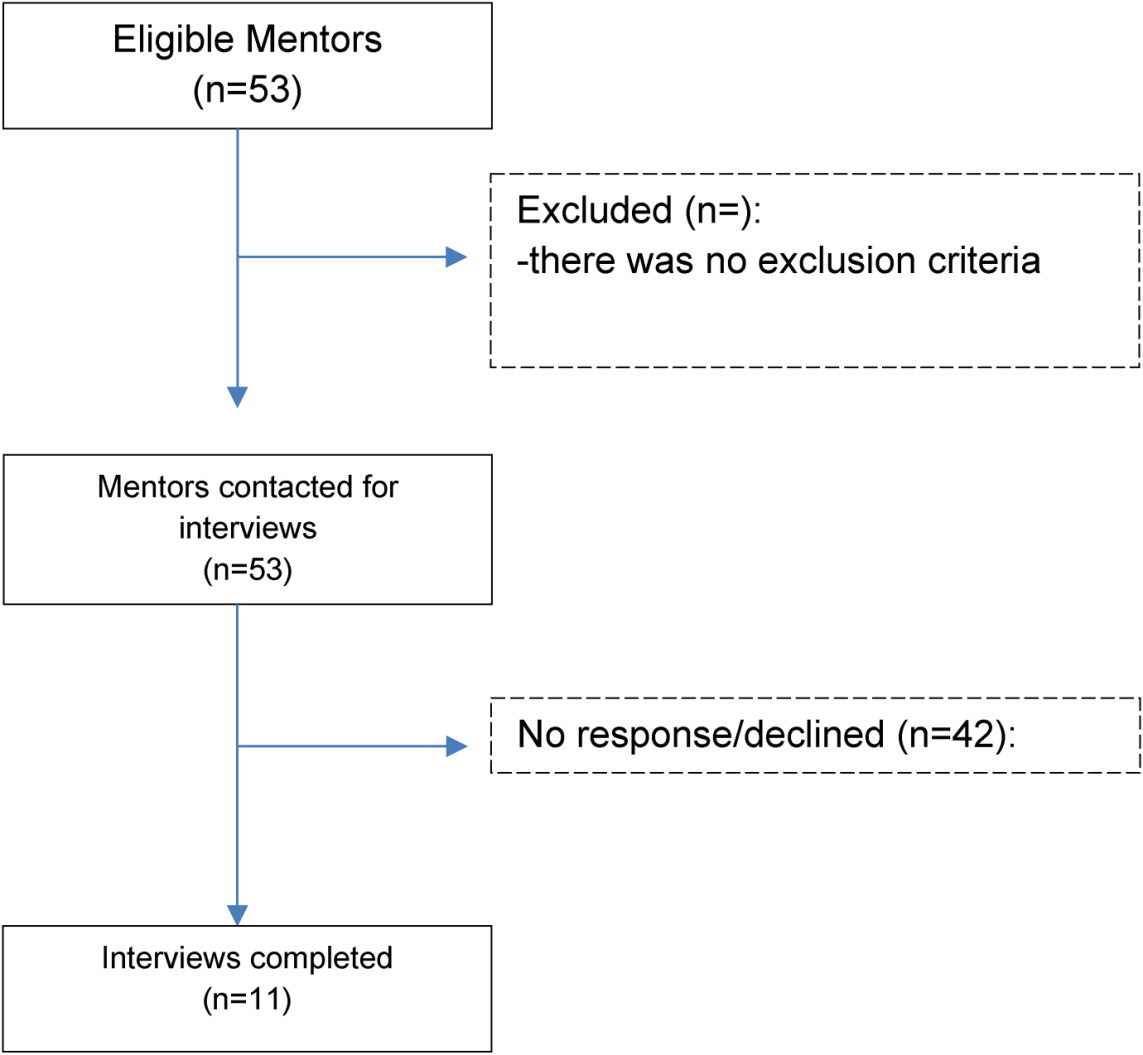
A COREQ (Consolidated criteria for Reporting Qualitative research) flow diagram for screening, contacting, and interviewing mentors.

Overall, 108 of 188 medical students (57.4%), and 11 of 53 mentors (20.7%), participated in the course evaluation. A COREQ (Consolidated Criteria for Reporting Qualitative Research) diagram for mentor interviews is illustrated in Figure 1. Tables are used to show results from student surveys (Table 2) and mentor interviews (Table 3), contextualized by narrative explanations and illustrations of key themes.^11^

**Table 2.** List of student survey self-efficacy statements, with pre-and post-course mean scores and p-values.

SELF-EFFICACY STATEMENT	MEAN PRE-COURSE RATING	MEAN POST-COURSE RATING	P-VALUE
**Leadership**			
I can easily adapt my leadership style to optimize group dynamics.	5.25	5.47	0.0001
If there is a conflict between members of my team, I am comfortable intervening.	5.06	5.26	0.0001
I make sure that my own reactions to stressful situations don't impact team performance.	5.47	5.67	<0.0001
I can apply self-management strategies to diffuse challenging situations.	5.30	5.44	0.0004
I am comfortable leading a group of my colleagues.	5.48	5.75	<0.0001
**Patient Safety and Quality Improvement**			
I can use a validated framework to analyze my clinical setting and identify a healthcare gap.	3.60	5.19	<0.0001
I am comfortable reporting issues of patient safety as they arise.	4.26	5.10	<0.0001
I am comfortable being the point person to assemble team members to address healthcare gaps.	3.86	4.65	<0.0001
I can quickly engage pertinent stakeholders to design quality improvement initiatives within my clinical setting.	3.34	4.56	<0.0001
I can effectively strategize to overcome resistance while implementing a quality improvement project.	3.56	4.53	<0.0001
I understand the difference in methodology between quality improvement and scientific research.	3.35	5.82	<0.0001
**Ethics & Social Accountability**			
I know my bias tendencies toward ethical issues in healthcare.	4.81	5.28	<0.0001
My personal biases will interfere with my ability to give equal care to all patients.	2.70	2.61	0.1411
I feel socially accountable in my future role as physician in the healthcare system.	5.53	5.91	<0.0001
I know how to identify resources to help manage challenging ethical scenarios in my workplace.	4.10	4.99	<0.0001
**Healthcare Systems & Finance**			
I know how the various provincial and national organizations (OMA, CMA, CMPA, PARO) will impact my future practice.	3.56	5.00	<0.0001
I have no trouble managing my personal finances.	4.45	4.58	0.0224
I know what it takes to manage my future professional finances.	3.59	4.17	<0.0001
I understand the role of healthcare funding in healthcare delivery.	4.19	5.17	<0.0001
I can analyze my healthcare system and compare it to other models of healthcare.	4.12	5.03	<0.0001

**Table 3.** List of key remarks from mentor interviews on students’ development in leadership and patient safety and quality improvement.

AREAS DEVELOPED	QUOTES
**Leadership**	*1. “This wasn't just an exercise, they actually became members or part of this team, for a brief period of time, but nonetheless, they were kind of a valued member there. I think it gave them a real practical experience. They were able to interact with RTs, nurses, surgeons, anesthesiologists, and intensivists.” -* Mentor 5 *2. “I saw the students really in there with the nitty-gritty of, like, getting to know the hospital network, getting to know the key people who mattered and understanding the nuances of the healthcare system.”* – Mentor 4 *3. “…They learn to work in a team, and they learn to tolerate and go on, or get along, with all different kinds of students.” –* Mentor 3 *4. “I think by the end, [students] were functioning together better.. they came together.. more integrated in the way they were doing things. I could see they were working a little bit more cohesively, the presentation was a joint effort.” –* Mentor 5 *5. “It definitely got a lot better towards the end as the more meetings we had the more we were able to bounce ideas off each other. Perhaps at the beginning I had to probe much more often and had to offer more insights and information in general whereas near the end I could pose a question to the team and let them work perhaps with more minor redirections and refinement so definitely improved through the year.” –* Mentor 9 *6. “I think that there were frustrations in the project I think that created some periods and valleys actually as we went through the year and what I mean by that is that expectations that the students had with how quickly things would happen how easily they would happen that sort of thing and I think they realized that quality improvement over the year is difficult and that it really does involves systems-based approach and system-based knowledge and there are problems with scoping and identifying stakeholders and all that that create fundamental issues in practically getting a project done and that created a lot of valleys for the students…”.* -Mentor 7 *7. “I think they appreciated that things that looked easy to do actually took way longer time in clinical studies, and that was the main things that things at first seemed simple and easy to do, and by the end of the year, they realized that because of how complicated the system is and how different individuals acted and stakeholders are involved they actually had to realize that things would take longer than anticipated.”* -Mentor 8 *8. “They came out with questions that we did think were good ways of looking at things. I should add that we worked on the antipsychotic polypharmacy project, so we are looking at gathering data, but it was the students who came up with the idea of introducing electronic reminders on powerchart to alert physicians, which was good. “ –* Mentor 11
**Patient Safety and Quality Improvement**	*1. “I did see some change in knowledge and attitudes over the year, and really overall, they were very positive changes, and I really saw how these sort of manifested themselves at the end of year as the students were presenting their project, so they were using vocabulary and terms that they had never used before, and they used them comfortably and appropriately, so that was good. They were also using tools, tools that most of them had never utilized before, and they were using these tools specifically to use in their project, and that was exciting to see.” –* Mentor 7 *2. “…by the end, they were familiar with the whole process of quality improvement.” –* Mentor 5 *3. “Lots of people were kind of skeptical about (it), or didn't see (value), because it's not research per se, but by the end, their reflections were: wow, this is a real thing, and it works, and I can see how it's practical, and it's a lot of work, but it's also not that much work kind of thing… So, for sure, I think it was the project in particular that worked through it, that allowed them to apply it, and not just kind of learn about it and say, ‘well, that's nice, and it's a theory’. It's actually practical. “ –* Mentor 6 *4. “I was quite impressed my team was able to delve into the literature and identify the issue we were addressing they were able to demonstrate an ability to come up with feasible and plausible solutions and were able to synthesize that into an initiative that was shovel ready so to speak and ready to implement.” –* Mentor 9 *5. “I was really impressed with just how motivated the students were in finding their own niche project, and really all I was there for was to help fine-tune things.” –* Mentor 4

### Leadership development

Our intervention positively impacted students’ comfort and confidence with leading groups. Students became more comfortable with *‘leading a group of my colleagues’* (Pre/post: 5.48/5.75, P < 0.0001) and with *‘being the point person to assemble team members to address healthcare gaps’* (Pre/post: 3.86/4.65, P < 0.0001). Students also self-reported growth in leadership, such as their ability to *‘adapt (their) leadership style to optimize group dynamics’* (Pre/post: 5.28/5.47, *P* = 0.0001). Furthermore, self-reported results indicate an improvement in their ability to ‘*engage pertinent stakeholders to design quality improvement initiatives within my clinical setting*’ (Pre/post: 3.34/4.56, *P* < 0.0001) and use ‘*a validated framework to analyze (a) clinical setting and identify a healthcare gap*’ (Pre/post: 3.60/5.19, *P* < 0.0001).

Mentor interviews also supported the course as a valuable leadership experience for students. Mentors commented on the course providing students with a practical clinical experience working with an interprofessional healthcare team (see Table 2 Leadership Quote 1 – LQ1), helping them understand the hospital network and the nuances of the healthcare system (LQ2). Students received first-hand experience leading diverse functional teams (LQ3), and the teams matured throughout their projects to develop synergy and effective team dynamics. Mentors found that “*by the end, [students] were functioning together better… they came together… more integrated in the way they were doing things.*” (LQ4). Some additional quotations by mentors include:

“*I think they appreciated that things that looked easy to do actually took way longer time in clinical studies, and that was the main things that things at first seemed simple and easy to do, and by the end of the year, they realized that because of how complicated the system is and how different individuals acted and stakeholders are involved they actually had to realize that things would take longer than anticipated.”* -Mentor 8 (LQ7)

“*They came out with questions that we did think were good ways of looking at things. I should add that we worked on the antipsychotic polypharmacy project, so we are looking at gathering data, but it was the students who came up with the idea of introducing electronic reminders on powerchart to alert physicians, which was good. “ –* Mentor 11 (LQ8)

### Patient safety and quality improvement

The WUPIC provided pre-clinical students with a unique, enriching opportunity to engage with PSQI projects that have practical implications in healthcare. Survey results suggest that students improved on PSQI knowledge, such as their understanding of *‘the difference in methodology between quality improvement and scientific research’* (Pre/post: 3.35/5.82, *P* < 0.0001) and became more ‘*comfortable reporting issues of patient safety as they arise’* (Pre/post: 4.26/5.10, *P* < 0.0001). Furthermore, a positive outcome of the course was that the project which received the highest overall evaluation (based on group presentation and written report) was later taken to full implementation in the PDSA cycle (after the completion of the course), and resulted in improvements to the home dialysis program at London Health Science Centre.

Our mentor interviews also indicated that the course was a valuable learning experience for PSQI. Mentors noted improvements in knowledge as students began “*using vocabulary and terms that they had never used before and using them comfortably and appropriately*” (See Table 2 Patient Safety Quality Improvement Quote 1 – PSQIQ1), and how by the end of their projects*,* they were “*familiar with the whole process of quality improvement*” (PSQIQ2). Furthermore, by the end of the course, students gained an appreciation and developed a positive attitude toward PSQI. For example, one mentor initially saw students being “*skeptical about (it), or didn't see (value), because it's not research per se*”. But by the end, “*their reflections were: wow, this is a real thing, and it works, and I can see how it's practical*” (PSQIQ3). Some additional quotes by mentors include:

“I was quite impressed my team was able to delve into the literature and identify the issue we were addressing they were able to demonstrate an ability to come up with feasible and plausible solutions and were able to synthesize that into an initiative that was shovel ready so to speak and ready to implement.” – Mentor 9 (PSQIQ4)

“I was really impressed with just how motivated the students were in finding their own niche project, and really all I was there for was to help fine-tune things.” – Mentor 4 (PSQIQ5)

## Discussion

Supporting the development of leadership and PSQI knowledge, skills, and attitudes in pre-clinical undergraduate training is indeed a tall order. The WUPIC allowed students to approach a real healthcare problem and to interact directly with an engaged faculty mentor. Experiential learning around leadership and PSQI requires students to see themselves as leading their own experiences, reflecting on them, developing self-awareness and determining what they want their future selves to look like. Our results support improvements in students’ knowledge, skills, and perceptions of leadership and PSQI. The significant improvement in quantitative self-assessment also could point to a general lack of both PSQI education and exposure in undergraduate medical education and underscores the importance and values of such educational interventions.

Our findings suggest that a key to implementing a similar undergraduate program is to design project-based faculty-mentored but student-led groups at the core of the curricular intervention. Faculty mentorship was feasible in this intervention despite the competing demands on faculty, as faculty engagement was scheduled judiciously (eg, monthly), PSQI expertise was not required, and role expectations were limited and defined. Additionally, our findings add to previous studies on the importance of mentorship for medical students,^12,13^ and the importance of students taking a lead role within the relationship.^14^

The PSQI projects were at the heart of the experiential learning of this course. These projects provided valuable leadership and PSQI experience through orienting them to a clinical setting, connecting students to stakeholders, and helping students brainstorm clinical gaps. A critical factor in the success of our intervention was the autonomy which the students’ groups were provided in leading their projects. While mentors were providing a supporting role, the students were in the driver's seat. This required students to work together in teams effectively and navigate new challenges that required collaboration and ingenuity. The nature of the projects forced the students to move beyond their comfort zones and engage with different stakeholders to come up with innovative solutions to challenges as they arose.

Medical schools that choose to invest in leadership and stewardship education in pre-clinical years stand to benefit on multiple levels. As students enter their clinical years, their first-hand leadership experience will serve them well in increasing their capacity and confidence to take on the leadership roles required of them as clerks and residents. They will also be well acquainted with the PSQI process that is increasingly integrated into clinical training.^15^ Many residency programs across North America have implemented PSQI projects as part of their core training requirements.^16–18^ Both the Accreditation Council for Graduate Medical Education (ACGME – the body responsible for accrediting all graduate medical training programs for physicians in the United States) & CanMEDS (a framework that provides a comprehensive foundation for medical education and practice in Canada), place patient safety, quality assurance, and systems-based learning as central to their competency frameworks, and as such giving students a head-start in becoming familiar and even proficient in this space, will carry many advantages as they enter residency programs.

The WUPIC has demonstrated that it is possible to provide undergraduate pre-clinical students with meaningful experiential leadership and PSQI education. The WUPIC has since undergone three successful academic cycles between 2016 and 2019, with a total of 75 unique mentored projects conducted during this time frame. In 2019, Western University underwent a curricular revamp to align with competency-based education and the core component of the WUPIC was transitioned into a new ‘experiential learning’ course that included all the core components of the WUPIC.

## Limitations of study

In spite of these positive outcomes, our course and research design were not without their limitations. A challenge for mentors was striking a balance between being overly involved in the project and giving students room to work through challenges themselves. Furthermore, the required number of mentors made it necessary to expand our selection to include faculty, fellows, and senior residents, which could have created varying student experiences. Limitations to our program evaluation include the student data being limited to student surveys, our interviews being limited to 11 mentors from a total of 53, and the data from one year of program implementation. There was no a priori calculation for sample size selected for the study, and all willing participants were included. Another limitation of our intervention is that the interview questions and self-efficacy rating tool were not piloted and validated previously. Lastly, only one of the projects in the course went through all stages of the Kirkpatrick model, and therefore another limitation of the study is that for almost all of the projects, our intervention only addressed the first two levels of the Kirkpatrick's model (levels 1 and 2), reaction and learning, but not behavior and results (levels 3 and 4).^19^ To improve future course assessment, future evaluations of the intervention should include a longitudinal evaluation, where prospective assessment of learner behavior is done in their clinical training years, and tracking of whether learners will continue to engage in PSQI activities in future years. An assessment of future leadership competencies during senior clinical training would also provide a useful assessment outcome.

## Conclusion

Interventions should give students a sense of ownership over their learning while providing the necessary support for optimal uptake and enhanced clinical competence. Given the effectiveness of our intervention, we encourage medical schools across North America to consider adopting faculty-mentored leadership and PSQI experiential learning opportunities in their undergraduate programs.

## Disclosures

## Supplemental Material

sj-pdf-1-mde-10.1177_23821205231170522 - Supplemental material for An Approach to Leadership Development and Patient Safety and Quality Improvement Education in the Context of Professional Identity Formation in Pre-Clinical Medical Students Click here for additional data file. Supplemental material, sj-pdf-1-mde-10.1177_23821205231170522 for An Approach to Leadership Development and Patient Safety and Quality Improvement Education in the Context of Professional Identity Formation in Pre-Clinical Medical Students by Hamza Inayat, Jacqueline Torti, Juliya Hemmett, Lorelei Lingard, Brandon Chau, Ali Inayat, Jason L. Elzinga and Nabil Sultan in Journal of Medical Education and Curricular Development

sj-pdf-2-mde-10.1177_23821205231170522 - Supplemental material for An Approach to Leadership Development and Patient Safety and Quality Improvement Education in the Context of Professional Identity Formation in Pre-Clinical Medical Students Click here for additional data file. Supplemental material, sj-pdf-2-mde-10.1177_23821205231170522 for An Approach to Leadership Development and Patient Safety and Quality Improvement Education in the Context of Professional Identity Formation in Pre-Clinical Medical Students by Hamza Inayat, Jacqueline Torti, Juliya Hemmett, Lorelei Lingard, Brandon Chau, Ali Inayat, Jason L. Elzinga and Nabil Sultan in Journal of Medical Education and Curricular Development

sj-pdf-3-mde-10.1177_23821205231170522 - Supplemental material for An Approach to Leadership Development and Patient Safety and Quality Improvement Education in the Context of Professional Identity Formation in Pre-Clinical Medical Students Click here for additional data file. Supplemental material, sj-pdf-3-mde-10.1177_23821205231170522 for An Approach to Leadership Development and Patient Safety and Quality Improvement Education in the Context of Professional Identity Formation in Pre-Clinical Medical Students by Hamza Inayat, Jacqueline Torti, Juliya Hemmett, Lorelei Lingard, Brandon Chau, Ali Inayat, Jason L. Elzinga and Nabil Sultan in Journal of Medical Education and Curricular Development
