# Supplementary material for: An Approach to Leadership Development and Patient Safety and Quality Improvement Education in the Context of Professional Identity Formation in Pre-Clinical Medical Students
Source: J Med Educ Curric Dev. 2023 May 8;10:23821205231170522. doi: 10.1177/23821205231170522 (PMC10176555; doi:10.1177/23821205231170522)
Supplement: sj-pdf-1-mde-10.1177_23821205231170522 - Supplemental material for An Approach to Leadership Development and Patient Safety and Quality Improvement Education in the Context of Professional Identity Formation in Pre-Clinical Medical Students [file sj-pdf-1-mde-10.1177_23821205231170522.pdf]

Professional Identity Self-Efficacy Survey

Please rate your agreement with the following statements. Ensure that you complete both "before" and "after" columns for each question. Supplemental comments can be provided in the space at the end of the survey. All responses will be anonymously aggregated to help us improve the course curriculum for future years, and will in no way impact individuals' academic standing. Thank you for participating!

Please indicate your campus:	Windsor ①	London ②

Leadership	Before the Professional Identity Course	After the Professional Identity Course
I can easily adapt my leadership style to optimize group dynamics.	Strongly Disagree — Neutral — Strongly Agree<br>① ② ③ ④ ⑤ ⑥ ⑦	Strongly Disagree — Neutral — Strongly Agree<br>① ② ③ ④ ⑤ ⑥ ⑦
If there is a conflict between members of my team, I am comfortable intervening.	Strongly Disagree — Neutral — Strongly Agree<br>① ② ③ ④ ⑤ ⑥ ⑦	Strongly Disagree — Neutral — Strongly Agree<br>① ② ③ ④ ⑤ ⑥ ⑦
I make sure that my own reactions to stressful situations don't impact team performance.	Strongly Disagree — Neutral — Strongly Agree<br>① ② ③ ④ ⑤ ⑥ ⑦	Strongly Disagree — Neutral — Strongly Agree<br>① ② ③ ④ ⑤ ⑥ ⑦
I can apply self-management strategies to diffuse challenging situations.	Strongly Disagree — Neutral — Strongly Agree<br>① ② ③ ④ ⑤ ⑥ ⑦	Strongly Disagree — Neutral — Strongly Agree<br>① ② ③ ④ ⑤ ⑥ ⑦
I am comfortable leading a group of my colleagues.	Strongly Disagree — Neutral — Strongly Agree<br>① ② ③ ④ ⑤ ⑥ ⑦	Strongly Disagree — Neutral — Strongly Agree<br>① ② ③ ④ ⑤ ⑥ ⑦

Quality Improvement & Patient Safety	Before the Professional Identity Course	After the Professional Identity Course
I can use a validated framework to analyze my clinical setting and identify a healthcare gap.	Strongly Disagree — Neutral — Strongly Agree<br>① ② ③ ④ ⑤ ⑥ ⑦	Strongly Disagree — Neutral — Strongly Agree<br>① ② ③ ④ ⑤ ⑥ ⑦
I am comfortable reporting issues of patient safety as they arise.	Strongly Disagree — Neutral — Strongly Agree<br>① ② ③ ④ ⑤ ⑥ ⑦	Strongly Disagree — Neutral — Strongly Agree<br>① ② ③ ④ ⑤ ⑥ ⑦
I am comfortable being the point person to assemble team members to address healthcare gaps.	Strongly Disagree — Neutral — Strongly Agree<br>① ② ③ ④ ⑤ ⑥ ⑦	Strongly Disagree — Neutral — Strongly Agree<br>① ② ③ ④ ⑤ ⑥ ⑦
I can quickly engage pertinent stakeholders to design quality improvement initiatives within my clinical setting.	Strongly Disagree — Neutral — Strongly Agree<br>① ② ③ ④ ⑤ ⑥ ⑦	Strongly Disagree — Neutral — Strongly Agree<br>① ② ③ ④ ⑤ ⑥ ⑦
I can effectively strategize to overcome resistance while implementing a quality improvement project.	Strongly Disagree — Neutral — Strongly Agree<br>① ② ③ ④ ⑤ ⑥ ⑦	Strongly Disagree — Neutral — Strongly Agree<br>① ② ③ ④ ⑤ ⑥ ⑦
I understand the difference in methodology between quality improvement and scientific research.	Strongly Disagree — Neutral — Strongly Agree<br>① ② ③ ④ ⑤ ⑥ ⑦	Strongly Disagree — Neutral — Strongly Agree<br>① ② ③ ④ ⑤ ⑥ ⑦

Ethics & Social Accountability	**Before the Professional Identity Course**	**After the Professional Identity Course**
I know my bias tendencies toward ethical issues in healthcare.	Strongly Disagree ① ② ③ ④ Neutral ⑤ ⑥ ⑦ Strongly Agree	Strongly Disagree ① ② ③ ④ Neutral ⑤ ⑥ ⑦ Strongly Agree
My personal biases will interfere with my ability to give equal care to all patients.	Strongly Disagree ① ② ③ ④ Neutral ⑤ ⑥ ⑦ Strongly Agree	Strongly Disagree ① ② ③ ④ Neutral ⑤ ⑥ ⑦ Strongly Agree
I feel socially accountable in my future role as physician in the healthcare system.	Strongly Disagree ① ② ③ ④ Neutral ⑤ ⑥ ⑦ Strongly Agree	Strongly Disagree ① ② ③ ④ Neutral ⑤ ⑥ ⑦ Strongly Agree
I know how to identify resources to help manage challenging ethical scenarios in my workplace.	Strongly Disagree ① ② ③ ④ Neutral ⑤ ⑥ ⑦ Strongly Agree	Strongly Disagree ① ② ③ ④ Neutral ⑤ ⑥ ⑦ Strongly Agree

Healthcare Systems & Finance	**Before the Professional Identity Course**	**After the Professional Identity Course**
I know how the various provincial and national organizations (OMA, CMA, CMPA, PARO) will impact my future practice.	Strongly Disagree ① ② ③ ④ Neutral ⑤ ⑥ ⑦ Strongly Agree	Strongly Disagree ① ② ③ ④ Neutral ⑤ ⑥ ⑦ Strongly Agree
I have no trouble managing my personal finances.	Strongly Disagree ① ② ③ ④ Neutral ⑤ ⑥ ⑦ Strongly Agree	Strongly Disagree ① ② ③ ④ Neutral ⑤ ⑥ ⑦ Strongly Agree
I know what it takes to manage my future professional finances.	Strongly Disagree ① ② ③ ④ Neutral ⑤ ⑥ ⑦ Strongly Agree	Strongly Disagree ① ② ③ ④ Neutral ⑤ ⑥ ⑦ Strongly Agree
I understand the role of healthcare funding in healthcare delivery.	Strongly Disagree ① ② ③ ④ Neutral ⑤ ⑥ ⑦ Strongly Agree	Strongly Disagree ① ② ③ ④ Neutral ⑤ ⑥ ⑦ Strongly Agree
I can analyze my healthcare system and compare it to other models of healthcare.	Strongly Disagree ① ② ③ ④ Neutral ⑤ ⑥ ⑦ Strongly Agree	Strongly Disagree ① ② ③ ④ Neutral ⑤ ⑥ ⑦ Strongly Agree

**General Comments**	
This course helped shape my professional identity as a future physician.	Strongly Disagree ① ② ③ ④ Neutral ⑤ ⑥ ⑦ Strongly Agree
I would recommend this course to my peers.	Strongly Disagree ① ② ③ ④ Neutral ⑤ ⑥ ⑦ Strongly Agree
Strengths of the Course:	
Suggestions for Improvement to the Course:	
