# Supplementary material for: An Approach to Leadership Development and Patient Safety and Quality Improvement Education in the Context of Professional Identity Formation in Pre-Clinical Medical Students
Source: J Med Educ Curric Dev. 2023 May 8;10:23821205231170522. doi: 10.1177/23821205231170522 (PMC10176555; doi:10.1177/23821205231170522)
Supplement: sj-pdf-2-mde-10.1177_23821205231170522 - Supplemental material for An Approach to Leadership Development and Patient Safety and Quality Improvement Education in the Context of Professional Identity Formation in Pre-Clinical Medical Students [file sj-pdf-2-mde-10.1177_23821205231170522.pdf]

Interview questions for mentors

Q1. Please describe your experience as a QI project mentor for the Professional Identity course including successes and challenges.

Q2. Tell me about any changes in knowledge and attitudes of the students in your mentorship group throughout the year.

Q3. How has the course been beneficial to your own learning?

Q4. How has this changed your comfort level with patient safety and quality improvement?

Q5. Do you have any suggestions for future course iterations?
